# Is there a weekend effect after hip fracture surgery? A study of 74,410 hip fractures reported to the Norwegian Hip Fracture Register

**DOI:** 10.1080/17453674.2019.1683945

**Published:** 2019-10-30

**Authors:** Andrea Boutera, Eva Dybvik, Geir Hallan, Jan-Erik Gjertsen

**Affiliations:** a Faculty of Medicine, University of Bergen, Bergen;; b The Norwegian Hip Fracture Register, Department of Orthopedic Surgery, Haukeland University Hospital, Bergen;; c Department of Clinical Medicine (K1), University of Bergen, Bergen, Norway

## Abstract

Background and purpose — The term “weekend effect” describes differences in outcomes between patients treated at weekends compared with weekdays. We investigated whether there is a weekend effect for the risk of reoperation and mortality after hip fracture surgery at Norwegian hospitals.

Patients and methods — We included data from 76,410 hip fractures in patients 60 years and older reported to the Norwegian Hip Fracture Register (NHFR) between 2005 and 2017. Cox survival analyses with adjustments for age, sex, ASA class, type of fracture, operating method, and waiting time from fracture to surgery were used to calculate the risk of reoperation and death after surgeries performed at weekends compared with surgeries performed on weekdays.

Results — The mean age for all patients was 82 years, and 71% were female. 73% of fractures occurred on weekdays (Monday to Friday) and 27% during weekends (Saturday and Sunday). 71% of fractures were operated on a weekday and 29% at a weekend. Slightly increased mortality was observed during the 2 first months after weekend admission with hip fracture (HR 1.08; 95% CI 1.03–1.14). This did not continue in subsequent months, but the initial effect of weekend presentation was still apparent at 1-year follow-up. Further, there was no difference in mortality between patients who were operated at a weekend and patients operated on a weekday. Neither were there any differences in the risk of reoperation between weekday and weekend when comparing day of fracture or day of surgery.

Interpretation — Patients who suffered a hip fracture during a weekend had slightly increased mortality in the first 2 months postoperatively. Whether the surgery was done on weekdays or at weekends did not affect mortality or the risk of reoperation.

The “weekend effect” describes the difference between patients admitted or treated at weekends compared with weekdays (Party et al. 1977). In an effort to understand and prevent adverse outcomes after hospital admission, several studies have studied the “weekend effect” in hip fractures (Smith et al. 2014, Thomas et al. 2014, Kristiansen et al. 2016, Nijland et al. 2017).

Early surgery for hip fracture patients has been shown to be associated with better outcome, but at weekends there may be less staffing, lower efficiency at the hospitals, longer waiting time, and surgeons with less experience (Daugaard et al. 2012, Mathews et al. 2016, Authen et al. 2018). Another aspect is that medical staff at weekends often may be less familiar with the patients under their care (Kent et al. 2016). Weekend admission and weekend surgery may therefore be a risk factor for increased mortality for hip fracture patients.

To ensure that hip fractures are treated within the time limits given in several recommendations, there is a need to operate these fractures also during weekends (Ranhoff et al. 2019). Several studies have reported higher mortality for hip fracture patients operated during weekends (Thomas et al. 2014, Kent et al. 2016, Kristiansen et al. 2016). Other studies have not found any weekend effect for hip fracture patients (Foss and Kehlet 2006, Boylan et al. 2015, Nandra et al. 2017, Sayers et al. 2017, Asheim et al. 2018). Therefore, we investigated whether there is a weekend effect for risks of reoperation and mortality after hip fractures, by using data from the Norwegian Hip Fracture Register (NHFR). Our hypothesis was that the mortality and risk of reoperation was greater for patients suffering a hip fracture or operated due to a hip fracture at a weekend compared with weekdays.

## Patients and methods

This retrospective observational study was performed using routinely collected data in the Norwegian Hip Fracture Register (NHFR). Since 2005 the NHFR has aimed to improve and assure the quality of hip fracture treatment in Norway (Gjertsen et al. 2008). The NHFR has provided a detailed picture of trends in care, particularly in respect of change in surgical techniques (Gjertsen et al. 2017, Johansen et al. 2017). After each hip fracture surgery, the surgeon fills in a standardized paper form that is sent to the register. The form gives information on the patient’s age, sex, ASA class, cognitive function, and unique personal identification number assigned to each inhabitant in Norway. Day of operation and fracture, delay to surgery, classification of the fracture, type of operation, cause and type of reoperation, information on implants, duration of surgery, and surgeon’s experience is also registered. In this way, the NHFR can monitor the outcome of primary operations, any subsequent reoperations, and mortality for hip fracture patients treated in Norwegian hospitals. Compared with the Norwegian Patient Registry the completeness of primary operations in the NHFR is 95% for hemi­arthroplasties and 88% for osteosyntheses (Furnes et al. 2019).

The patients were categorized according to the ASA score system. Further, we divided patients into the following age categories: 60–74 years, 75–79, 80–84, 85–89, and over 90 years. Cognitive impairment was classified as: yes, no, or uncertain. Less than half of the cases in the NHFR had information on the exact time of fracture, but almost all cases had the date of fracture and operation registered. Therefore, we chose to define each day of the week as full days from 00–24 hours. “Weekdays” were defined as Monday through Friday and “Weekends” as Saturday and Sunday. One could argue that the weekend starts on Friday afternoon/evening, but at most Norwegian hospitals the staffing on Friday afternoon/evening is exactly the same as on any other “Weekday” afternoon/evening. This simple definition of “Weekdays” and “Weekends” can therefore be justified.

Surgeon’s experience has been registered in the NHFR since 2011. Surgeon’s experience was divided into 3 groups: less than 3 years’ experience, more than 3 years’ experience, and missing information on surgeons’ experience. Waiting time from fracture to surgery was identified by 5 groups: 0–6 hours, > 6–12 hours, > 12–24 hours, > 24–48 hours, and > 48 hours.

**Figure 1.**
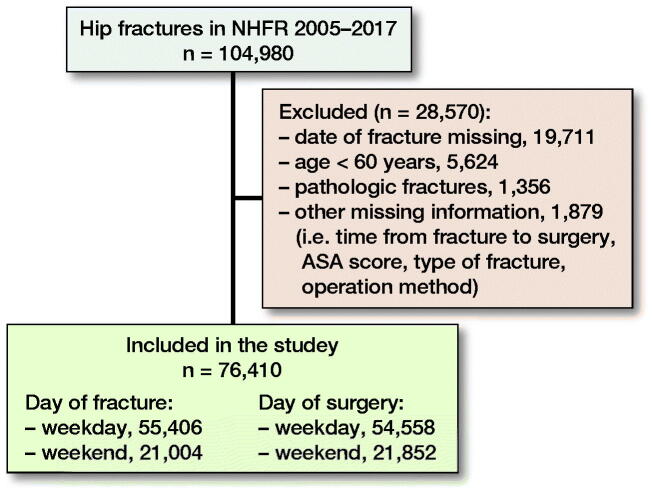
Patient selection.

As of December 31, 2017, the NHFR had information on 104,980 hip fractures treated between 2005 and 2017 (Figure 1). We excluded operations with missing data on day of fracture, ASA class, type of fracture, operation method, and time interval from fractures to surgery. Further, operations in patients under 60 years and operations of pathological fractures were excluded to get a more homogeneous patient group. After exclusion, 76,410 hip fractures remained for analyses. Of these, 55,406 (73%) hip fractures occurred on weekdays and 21,004 (27%) occurred during weekends. 54,558 (71%) hip fractures were operated on weekdays and 21,852 (29%) at weekends.

### Statistics

A chi-square test was used to compare means for categorical variables. Survival analyses were performed using Kaplan–Meier and Cox regression methods. Follow-up time was calculated from the index operation until the first reoperation, emigration, death, or December 31, 2018 (end of study), whichever came first. Cox multiple regression models were used to compare hazard ratios (HRs) for reoperation and death among patient groups (weekdays/weekends). Adjustments were done for age groups, sex, ASA class, type of fracture, operation method, and time from fracture to surgery. These adjustments were done as earlier studies on similar patient populations in the NHFR have shown that these specific variables may influence mortality and risk for reoperation. Adjustments for patients operated on both sides were not done, since an earlier study has shown that this will not alter the conclusion for the entered covariates (Lie et al. 2004).

Reoperation and mortality were studied within 30, 60, 180, and 365 days after surgery. Further, sub-analyses using a more common definition of “Weekend” (Friday 4 pm–Monday 8 am) were done for patients with information on exact time of fracture.

P-values < 0.05 were considered statistically significant. Proportionality assumptions were checked using log minus log plots and these were fulfilled. The statistical analyses were performed using IMB-SPSS Statistics, version 24.0 for Windows (IBM Corp, Armonk, NY, USA) and the statistical package R, version 3.4.0 (http://www.R-project.org). The study was performed in accordance with the RECORD statement.

### Ethics, funding, and potential conflicts of interest

The NHFR has permission from the Norwegian Data Protection Authority to collect and store data on hip fracture patients (permission issued January 3, 2005; reference number 2004/1658-2 SVE/-). The patients have signed a written, informed consent, and in case they were not able to understand or sign, their next of kin could sign the consent form on their behalf. The Norwegian Hip Fracture Register is financed by the Western Norway Regional Health Authority. No competing interests were declared.

## Results

### Patients

The mean age of all patients was 82 years, and 71% were female. About 25% of the study population had cognitive impairment, and 62% of all patients were ASA class 3 or higher. There were small differences in baseline characteristics between the treatment groups (Table 1). Operation data for day of fracture and day of surgery showed only small statistically significant differences in type of primary operation and waiting time (Table 2).

**Table 1.** Baseline characteristics for all patients with separate numbers for day of fracture and day of surgery. Values are frequency (%)

	Day of fracture			Day of surgery		
Characteristics	Weekday	Weekend	p-value ^a^	Weekday	Weekend	p-value ^a^
Total (76,410)	55,406	21,004		54,558	21,852	
Age category			0.003			< 0.001
	60–74	9,603 (17)	3,873 (19)		9,464 (17)	4,012 (18)
	75–79	7,873 (14)	2,950 (14)		7,717 (14)	3,106 (14)
	80–84	12,536 (23)	4,569 (22)		12,247 (23)	4,858 (22)
	85–89	14,272 (26)	5,399 (26)		14,087 (26)	5,584 (26)
	≥ 90	11,122 (20)	4,213 (20)		11,043 (20)	5,584 (20)
Female sex	39,452 (71)	14,978 (71)	0.3	38,817 (71)	15,513 (71)	0.3
ASA classification			0.1			0.001
	1	2,300 (4.2)	941 (4.5)		2,251 (4.1)	990 (4.5)
	2	18,839 (34)	7,195 (34)		18,518 (34)	7,516 (34)
	3	30,367 (55)	11,472 (55)		29,901 (55)	11,938 (55)
	4	3,818 (6.9)	1,369 (6.5)		3,804 (7.0)	1,383 (6.3)
	5	82 (0.1)	27 (0.1)		84 (0.2)	25 (0.1)
Cognitive impairment			0.2			0.01
	No	35,472 (64)	13,398 (64)		34,698 (64)	14,172 (65)
	Yes	13,687 (25)	5,313 (25)		13,704 (25)	5,296 (24)
	Uncertain	5,252 (9.5)	1,938 (9.2)		5,189 (9.5)	2,001 (9.2)
	Missing	995 (1.8)	355 (1.7)		967 (1.8)	383 (1.8)
Type of fracture			0.7			0.6
	Undisplaced FNF	8,168 (15)	3,063 (15)		8,104 (15)	3,127 (14)
	Displaced FNF	22,679 (41)	8,752 (42)		22,396 (41)	9,035 (41)
	Basocervical FNF	2,004 (3.6)	718 (3.4)		1,952 (3.6)	770 (3.5)
	Trochanteric					
	AO/OTA A1	9,414 (17)	3,552 (17)		9,250 (17)	3,716 (17)
	AO/OTA A2	8,829 (16)	3,309 (16)		8,651 (16)	3,487 (16)
	AO/OTA A3	940 (1.7)	349 (1.7)		923 (1.7)	366 (1.7)
	Subtrochanteric	2,906 (5.2)	1,079 (5.1)		2,833 (5.2)	1,152 (5.3)
	Other	466 (0.8)	182 (0.9)		449 (0.8)	199 (0.9)

a Pearson’s chi-square test.

FNF = femoral neck fracture.

**Table 2.** Operative data with separate numbers for day of fracture and day of surgery. Values are frequency (%)

	Day of fracture			Day of surgery			
Characteristics	Weekday	Weekend	p ^a^	Weekday	Weekend	p ^a^	
Total (76,410)	55,406	21,004		54,558	21,852		
Surgeon’s experience level ^b^			0.5			1.0	
	< 3 years	4,680 (16)	1,789 (16)		4,612 (16)	1,857 (16)	
	≥ 3 years	22,375 (79)	8,591 (79)		22,085 (79)	8,881 (79)	
	Missing	1,450 (5.1)	522 (4.8)		1,407 (5.0)	565 (5.0)	
Time from fracture to operation			< 0.001			< 0.001	
	0–6 hours	2,579 (4.7)	1,197 (5.7)		2,614 (4.8)	1,162 (5.3)	
	> 6–12 hours	9,266 (17)	3,441 (16)		9,200 (17)	3,507 (16)	
	> 12–24 hours	20,190 (36)	7,807 (37)		19,414 (36)	8,583 (39)	
	> 24–48 hours	15,970 (29)	5,675 (27)		15,628 (29)	6,017 (28)	
	> 48 hours	7,401 (13)	2,884 (14)		7,702 (14)	2,583 (12)	
Type of primary operation			0.7			0.2	
	Screw osteosynthesis	10,968 (20)	4,246 (20)		10,862 (20)	4,352 (20)	
	Hemiprosthesis	19,734 (36)	7,524 (36)		19,452 (36)	7,776 (36)	
	Sliding hip screw	11,712 (21)	4,408 (21)		11,493 (21)	4,627 (21)	
	Sliding hip screw + TSP	5,021 (9.1)	1,863 (8.9)		4,855 (8.9)	2,029 (9.3)	
	Short IM nail	4,604 (8.3)	1,710 (8.1)		4,535 (8.3)	1,779 (8.1)	
	Long IM nail	2,371 (4.3)	896 (4.3)		2,383 (4.4)	884 (4.0)	
	Other	996( 1.8)	357 (1.7)		948 (1.7)	405 (1.9)	

a Pearson’s chi-square test.

b Registered since 2011.

TSP = trochanteric support plate; IM = intramedullary.

### Mortality and reoperations

Patients sustaining a hip fracture during weekends had 0.3–0.7% higher mortality than patients sustaining a hip fracture during weekdays (Figure 2, Table 3, see Supplementary data). Increased mortality was observed during the 2 first months after weekend admission with hip fracture (HR 1.03). This did not continue in subsequent months, but this initial effect of weekend presentation was still apparent at 1-year follow-up. Mortality was, however, independent of the day of surgery (Figure 3, Table 3, see Supplementary data). The risk of reoperation was independent of the day of injury and the day of surgery (Table 4, see Supplementary data). Analyses including only patients with information on exact time of fracture, and using a more common definition of “Weekend” (Friday 4 pm–Monday 8 am), gave similar results regarding both mortality and reoperations. Sub-analyses also showed that risks of reoperation and mortality after 1 year for technically demanding fractures (displaced femoral neck fractures and intertrochanteric/subtrochanteric fractures) and technically demanding surgeries (hemiarthroplasties and long IM nails) were independent of day of fracture as well as day of surgery (Table 5, see Supplementary data). Further, analyses showed that both healthy (ASA 1–2) patients (HR 1.08, 95% CI 1.00–1.16) and comorbid (ASA 3–5) patients (HR 1.04, 95% CI 1.00–1.08) with hip fracture occurring during the weekend had a small, but statistically significant increased mortality, but no increased risk of reoperations. Further, patients aged 80–89 years had increased risk of death when sustaining a fracture during weekends (HR 1.09, CI 1.04–1.14), but no increased risk of reoperations compared with fracture on weekdays. For the patients aged 60–79 years and ≥ 90 years there were no differences in mortality or reoperation risk when investigating either day of fracture or day of surgery.

Figure 2.30-day (left panel) and 1-year (right panel) mortality, grouped after time of fracture. Cox survival curves adjusted for differences in age, sex, ASA class, type of fracture, operation method and time from fracture to surgery.

**Figure:**
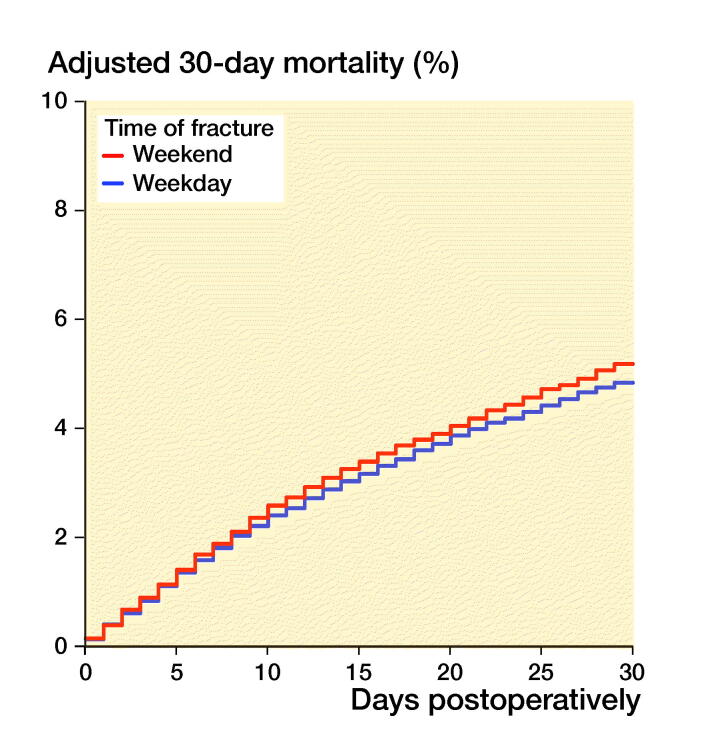

**Figure:**
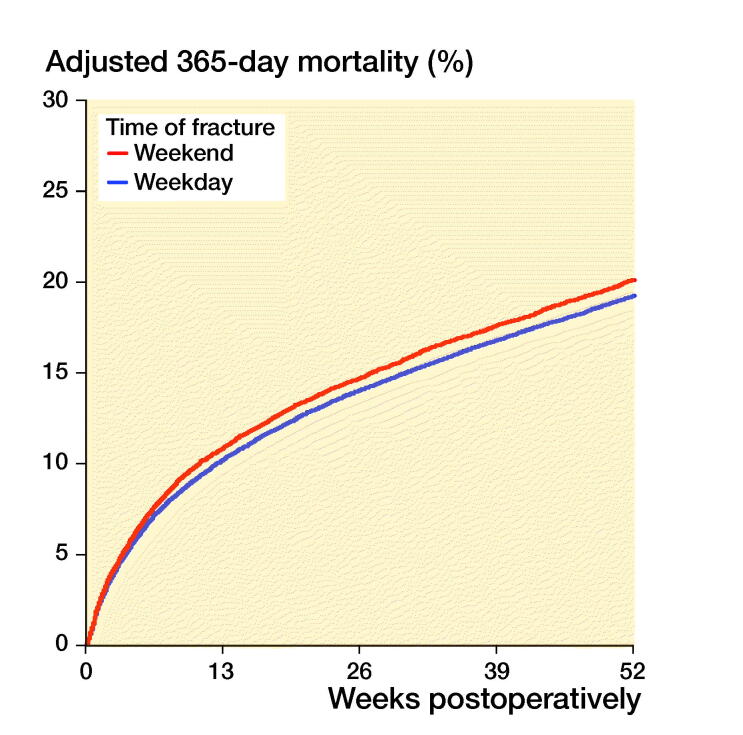

Figure 3.30-day (left panel) and 1-year (right panel) mortality, grouped on after time of surgery. Cox survival curves adjusted for differences in age, sex, ASA class, type of fracture, operation method and time from fracture to surgery.

**Figure:**
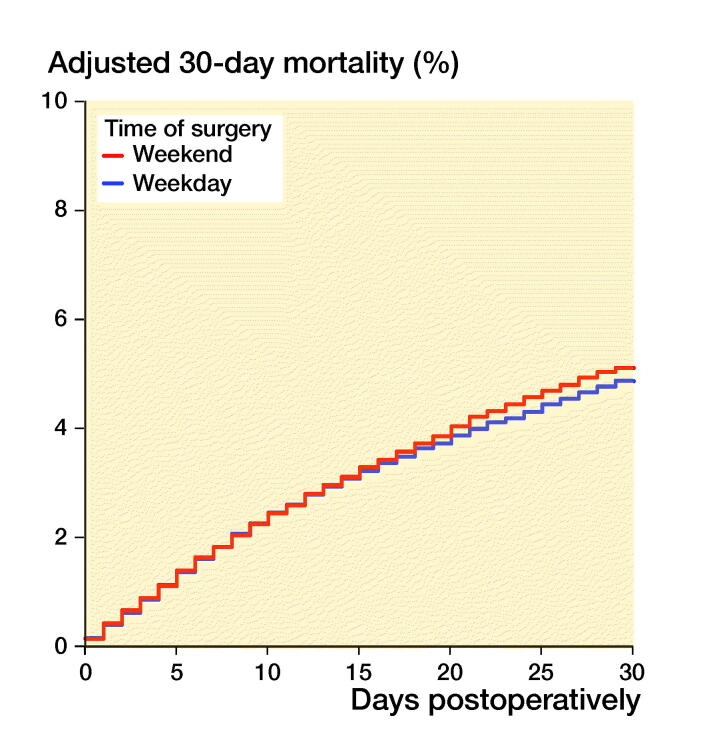

**Figure:**
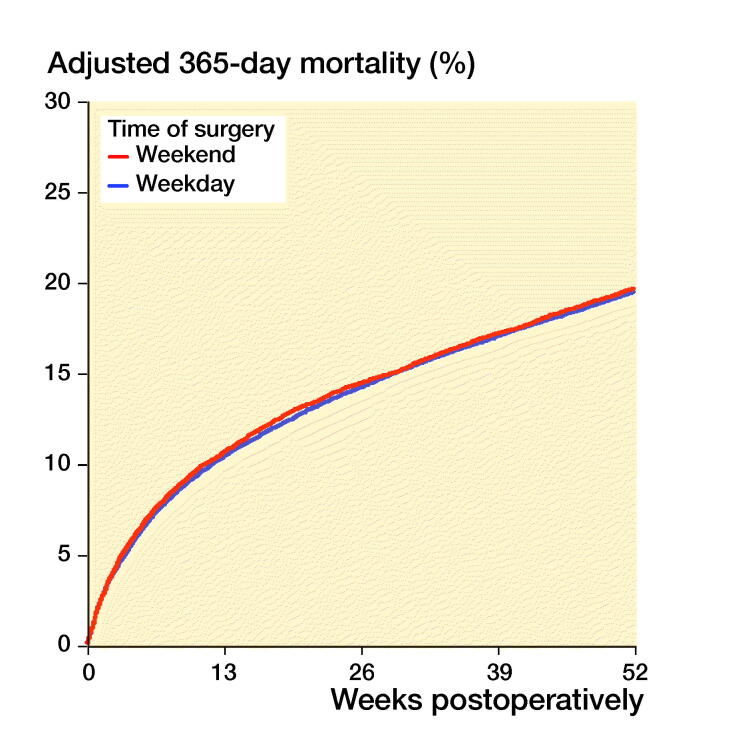

## Discussion

Overall, our results demonstrate that hip fracture patients at Norwegian hospitals had the same outcomes after surgery regardless of whether surgery was performed on a weekday or at a weekend. This is in line with several studies reporting on various outcomes (Foss and Kehlet 2006, Boylan et al. 2015, Mathews et al. 2016, Nandra et al. 2017, Nijland et al. 2017, Sayers et al. 2017, Asheim et al. 2018).

National guidelines for treatment of hip fracture patients are currently used at most hospitals in Norway (Ranhoff et al. 2019). The NICE guidelines recommend surgery on the same day or the day after the fracture (National Institute for Health and Clinical Excellence 2011). These guidelines contribute to standardized treatment of hip fracture patients on weekdays and at weekends. Thomas et al. (2014) found no differences in 30-day mortality rates between weekdays and weekends in a retrospective review of 2,989 patients with hip fractures. Another study with 1,326 patients demonstrated that specific outcomes, including length of stay, delay to surgery, longer-term mortality, and reoperation rates, did not differ between weekdays and weekend admission or surgery (Sheikh et al. 2018). Our results, and results from the UK, did not show a weekend effect; this implies that guidelines can be helpful.

Other factors than weekend surgery probably influence the mortality in hip fracture patients more. Some studies have suggested that surgical delay is one of the most important factors affecting the mortality for hip fracture patients (Daugaard et al. 2012, Sayers et al. 2017). We found a small difference in delay to surgery in baseline characteristics of patients.

We identified a slightly increased mortality in the first 2 months postoperatively for fractures during weekends, which may suggest that the quality of medical treatment during weekends is poorer compared with weekdays. Lower levels of nursing have been shown to be associated with increased mortality (Cram et al. 2004, Pauls et al. 2017). Thus, a decreased quality of perioperative treatment of hip fracture patients during weekends can explain the differences found in our study. One of the key factors in a well-run hip fracture unit is the multidisciplinary team input, particularly from the orthogeriatric service (Thomas et al. 2014). Patients sustaining a fracture during the weekend may have delayed orthogeriatric assessment. In addition, delays in community service like home care, at a nursing home, and in emergency rooms could affect time from fracture to admission to hospital during weekends.

In addition to reduced staff quantity at weekends, the weekend medical staff can be less experienced and less familiar with the patients on the ward (Kent et al. 2016). Less experienced staffing could cause a delay in preparation of hip fracture patients for the theatre as well. Reduced staffing at weekends also lowers the surgical capacity. The overall increase in difficulties in accessing subspecialty opinions, access to past medical records, less staffing, delay to surgery, and less experienced medical staff can probably all contribute to the increase in mortality for weekend fractures (Thomas et al. 2014).

A recently published study on the weekend effect for hip fracture treatment in Norway based on data from the Norwegian Patient Register (NPR) found slightly increased mortality for patients admitted on Sundays and during holidays, but the most remarkable result was that early morning admission and weekend discharge increased the mortality rate (Asheim et al. 2018). Other studies have also reported increased mortality for patients admitted during holidays, but not during weekdays and weekends (Foss and Kehlet 2006, Asheim et al. 2018).

The strength of our study is the large number of patients included in a national register with high completeness and relatively long patient follow-up. The fact that the study presents national results increases the external validity of this study.

Register-based studies do, however, have limitations including selection bias, information bias, and confounding (Varnum et al. 2019). We adjusted for possible confounding variables. The experience level of the surgeon was classified according to years of experience. However, it is likely that this classification does not necessarily reflect the volume of hip fracture operations that a surgeon has performed. In our study 27% of cases were excluded for different reasons, of which missing data constituted 21%. Incomplete reporting of reoperations may occur, but there is no reason to suspect a difference in reporting between surgeries or fractures on weekdays compared with weekends.

With the large number of patients in our study small differences between groups may reach statistical significance without being of clinical importance. Therefore, the clinical importance needs to be considered for all results presented.

In conclusion, patients who sustained a hip fracture during a weekend demonstrated a somewhat higher mortality in the first two months postoperatively compared with weekdays in our study. Other authors have proposed that the overall quality of treatment is somewhat poorer during weekends. This emphasizes the importance of optimal perioperative treatment of hip fracture patients during weekends too. Operating hip fractures during weekends did not increase mortality or risk of reoperations. Accordingly, no clear weekend effect could be found at Norwegian hospitals in this study.

## Supplementary data

Tables 3–5 are available as supplementary data in the online version of this article, http://dx.doi.org/10.1080/17453674.2019.1683945

## Supplementary Material

Supplemental Material
