# Supplementary material for: Is there a weekend effect after hip fracture surgery? A study of 74,410 hip fractures reported to the Norwegian Hip Fracture Register
Source: Acta Orthop. 2019 Oct 30;91(1):63–8. doi: 10.1080/17453674.2019.1683945 (PMC7008236; doi:10.1080/17453674.2019.1683945)
Supplement: Supplemental Material [file IORT_A_1683945_SM6062.pdf]

Supplementary data

Table 3. Number of deaths and hazard ratios (HRs) of death after fracture and operation at weekend relative to weekday

Mortality	Weekday (ref) Deaths n (%)	Weekend Deaths n (%)	Cox regression analysis [a] HR (95% CI)
30-day			
Day of fracture	3,856 (7.0)	1,541 (7.3)	1.07 (1.01–1.14)
Day of surgery	3,838 (7.1)	1,559 (7.0)	1.05 (0.99–1.11)
60-day			
Day of fracture	5,743 (10	2,316 (11)	1.08 (1.03–1.14)
Day of surgery	5,745 (11)	2,314 (10)	1.04 (0.99–1.09)
61- to 365-day			
Day of fracture	6,263 (13)	2,376 (13)	1.03 (0.98–1.08)
Day of surgery	6,241 (13)	2,398 (13)	0.98 (0.94–1.03)
180-day			
Day of fracture	9,305 (17)	3,644 (17)	1.05 (1.02–1.10)
Day of surgery	9,261 (17)	3,688 (17)	1.02 (0.99–1.06)
1-year			
Day of fracture	12,313 (22)	4,814 (23)	1.05 (1.02–1.09)
Day of surgery	12,276 (23)	4,851 (22)	1.01 (0.98–1.05)

[a] Cox regression analysis adjusted for age, sex, ASA class, type of fracture, operation method, and time from fracture to surgery.

Table 4. Number of reoperations and hazard ratios (HRs) of reoperations after fracture and operation at weekend relative to weekday

Reoperation within	Weekday (ref) Reoperation n (%)	Weekend Reoperation n (%)	Cox regression analysis [a] HR (95% CI)
30 days			
Day of fracture	2,458 (4.4)	962 (4.6)	1.03 (0.92–1.20)
Day of surgery	1,076 (2.0)	422 (1.9)	0.98 (0.87–1.09)
60 days			
Day of fracture	1,584 (2.9)	629 (3.0)	1.05 (0.96–1.15)
Day of surgery	1,582 (2.9)	631 (2.9)	1.00 (0.91–1.09)
180 days			
Day of fracture	2,458 (4.4)	962 (4.6)	1.03 (0.96–1.11)
Day of surgery	2,417 (4.4)	1,003 (4.6)	1.03 (0.96–1.11)
1 year			
Day of fracture	3,101 (5.6)	1,183 (5.6)	1.01 (0.94–1.08)
Day of surgery	3,017 (5.5)	1,267 (5.8)	1.04 (0.98–1.11)

[a] Cox regression analysis adjusted for age, sex, ASA class, type of fracture, operation method, and time from fracture to surgery.

Table 5. Number and hazard ratios (HRs) of reoperations within 1 year (day of surgery): sub-analyses of technical difficult fractures and surgeries

Reoperation	Weekday (ref) Reoperation n (%)	Weekend Reoperation n (%)	Cox regression analysis [a] HR (95% CI)
Difficult fractures			
Displaced FNF	1,337 (5.9)	578 (6.5)	1.06 (0.96–1.17)
Intertrochanteric/ subtrochanteric fracture	194 (5.2)	84 (5.5)	1.07 (0.83–1.39)
Difficult surgeries			
Hemiarthroplasty	749 (3.8)	315 (4.1)	1.05 (0.92–1.20)
Long IM nail	100 (4.0)	36 (4.5)	0.95 (0.65–1.40)

[a] Cox regression analysis adjusted for age, sex, ASA class, type of fracture, operation method and time from fracture to surgery.
FNF = femoral neck fracture; IM = intramedullary.
